# Modulation of atypical brain activation during executive functioning in autism: a pharmacological MRI study of tianeptine

**DOI:** 10.1186/s13229-021-00422-0

**Published:** 2021-02-19

**Authors:** Robert H. Wichers, James L. Findon, Auke Jelsma, Vincent Giampietro, Vladimira Stoencheva, Dene M. Robertson, Clodagh M. Murphy, Sarah Blainey, Grainne McAlonan, Christine Ecker, Katya Rubia, Declan G. M. Murphy, Eileen M. Daly

**Affiliations:** 1 Department of Forensic and Neurodevelopmental Sciences, The Sackler Centre for Translational Neurodevelopment, Institute of Psychiatry, Psychology and Neuroscience, King’s College London, PO50 De Crespigny Park, Denmark Hill, London, SE5 8AF UK; 2 Behavioural and Developmental Psychiatry Clinical Academic Group, South London and Maudsley NHS Trust, London, UK; 3 VU University Medical Center, Amsterdam, The Netherlands; 4 Department of Neuroimaging, Institute of Psychiatry, Psychology and Neuroscience, King’s College London, London, UK; 5 Department of Child and Adolescent Psychiatry, Psychosomatics and Psychotherapy, University Hospital Frankfurt am Main, Goethe-University, Frankfurt am Main, Germany; 6 Department of Child and Adolescent Psychiatry, Institute of Psychiatry, Psychology and Neuroscience, King’s College London, London, UK

**Keywords:** Autism spectrum disorder, Executive functioning, Tianeptine, Serotonin, fMRI

## Abstract

**Background:**

Autism spectrum disorder (ASD) is associated with deficits in executive functioning (EF), and these have been suggested to contribute to core as well as co-occurring psychiatric symptoms. The biological basis of these deficits is unknown but may include the serotonergic system, which is involved both in regulating EF in neurotypical populations and in the pathophysiology of ASD. We previously demonstrated that reducing serotonin by acute tryptophan depletion (ATD) shifts differences in brain function during performance of EF tasks towards control levels. However, ATD cannot be easily used in the clinic, and we therefore need to adopt alternative approaches to challenge the serotonin system. Hence, we investigated the role of the serotonergic modulator tianeptine on EF networks in ASD.

**Method:**

We conducted a pharmacological magnetic resonance imaging study, using a randomized double-blind crossover design, to compare the effect of an acute dosage of 12.5 mg tianeptine and placebo on brain activation during two EF tasks (of response inhibition and sustained attention) in 38 adult males: 19 with ASD and 19 matched controls.

**Results:**

Under placebo, compared to controls, individuals with ASD had atypical brain activation in response inhibition regions including the inferior frontal cortex, premotor regions and cerebellum. During sustained attention, individuals with ASD had decreased brain activation in the right middle temporal cortex, right cuneus and left precuneus. Most of the case–control differences in brain function observed under placebo conditions were abolished by tianeptine administration. Also, within ASD individuals, brain functional differences were shifted significantly towards control levels during response inhibition in the inferior frontal and premotor cortices.

**Limitations:**

We conducted a pilot study using a single dose of tianeptine, and therefore, we cannot comment on long-term outcome.

**Conclusions:**

Our findings provide the first evidence that tianeptine can shift atypical brain activation during EF in adults with ASD towards control levels. Future studies should investigate whether this shift in the biology of ASD is maintained after prolonged treatment with tianeptine and whether it improves clinical symptoms.

## Introduction

Autism spectrum disorder (ASD) is a complex, heterogeneous, neurodevelopmental condition with an estimated population prevalence of ~ 1:68 [1]. The cognitive phenotype of ASD includes atypical executive functioning (EF) [2, 3], which comprises a range of cognitive processes that are necessary for concentrating and paying and/or switching attention [4]. It has been suggested that core ASD symptoms may contribute to alterations in EF, including response inhibition and sustained attention. For example, restricted, stereotyped and repetitive behaviours (RSRBs) have been associated with abnormalities in inhibitory control [5], which have frequently been reported in ASD [6]. Abnormalities in sustained attention networks are also thought to at least partially underpin both core (e.g. communication) [7] and associated (e.g. attention deficit hyperactivity disorder (ADHD)) ASD symptoms [7]. Targeting EF networks may therefore be of clinical value in treating core and associated symptoms in ASD.

Brain regions that are activated during EF tasks have been implicated in ASD. For example, functional differences in the inferior and orbitofrontal cortex, caudate, thalamus and cerebellum have been reported in children and adults with ASD as compared to typically developing control subjects during response inhibition tasks [8–10], which has been further confirmed in a recent meta-analysis of functional MRI (fMRI) studies of cognitive control [11]. Also, fMRI studies using sustained attention tasks have reported significantly less activation in children and adults with ASD [7, 12] in regions associated with sustained attention, including the inferior and middle frontal, parietal, striato-thalamic and cerebellar regions [13, 14]. The biological basis of these differences is unknown, but the serotonergic system may be involved. For example, in neurotypical populations, increasing brain serotonin levels with a selective serotonin reuptake inhibitor (SSRI) [15] has been shown to improve performance on a response inhibition task, but also to impair sustained attention [16] and to reduce brain activation in frontal and subcortical attention networks during a vigilance task [17].

The serotonergic system has also been implicated in the pathophysiology of ASD by prior genetic, biochemical and neuroimaging studies. For example, SLC6A4 (the serotonin transporter gene) has been linked to the diagnosis of ASD [18] and hyperserotonemia has been observed in approximately 30% of ASD individuals [19]. Neuroimaging studies have reported a significant reduction in cortical 5-HT2A receptor density [20] and in the binding of the serotonin transporter in adults with ASD [21]. In addition, more recent evidence for the role of serotonin in modulating EF in ASD includes a report that abnormal brain activation during performance of a Go/No-Go task was shifted significantly towards control levels after reducing serotonin by acute tryptophan depletion (ATD) [9]. Moreover, the degree of change in brain activation correlated with the severity of RSRBs, suggesting a potential treatment application. However, ATD is an experimental procedure that cannot easily be used in a routine clinical setting. Thus, repurposing a drug that reduces serotonin may provide a novel treatment opportunity that could be (relatively) quickly ‘translated’ to the clinic.

Tianeptine has been reported to, among other actions, enhance the reuptake of serotonin [22, 23] and to have cognitive enhancing abilities [24, 25]. Hence, we tested the impact of tianeptine on brain function during an inhibitory and sustained attention task in ASD. Based on the study of ATD in adults with ASD [9] and tianeptine’s effect on cognitive functions, we hypothesized that abnormalities in brain activation during a Go/No-Go task of response inhibition and a sustained attention task, as measured by functional MRI, would be abolished in the ASD group after a single dose of tianeptine. We further hypothesized that the degree of responsivity would be related to severity of core or associated symptoms. Therefore, we aimed to provide proof of concept that a single dose of tianeptine can shift atypical brain activation in ASD towards a more typical profile.

## Materials and methods

### Participants

Nineteen male, right-handed adults with ASD and 19 typically developed (TD) control participants were included in the study (age: ASD mean = 30, SD = 11, TD mean = 27, SD = 9). Two ASD cases and 2 TD controls were excluded from the Go/No-Go task due to significant head movement, leaving a sample of 17 ASD cases and 17 TD controls for the Go/No-Go task, and 19 ASD cases and 19 TD controls for the sustained attention task. The sample size was chosen based on results from our prior experiments targeting serotonin modulation using acute tryptophan depletion [9, 26], which were successful in detecting group differences in BOLD response with sample sizes of *n* = 14. This implies an effect size (expressed in Cohen’s d) in excess of 1.2 [9, 26]. Exclusion criteria included medical disorders that could influence cognitive performance, major mental illnesses other than ASD, genetic disorders associated with ASD, alcohol or substance dependence or taking any medication affecting the serotonergic system (e.g. antidepressants, antipsychotics, benzodiazepines or mood stabilizers). The ASD diagnoses were made by a consultant psychiatrists using ICD-10 research criteria [27] and confirmed using the Autism Diagnostic Interview-Revised (ADI-R) [28] if an informant was available. Current autistic symptoms were measured by the Autism Diagnostic Observation Schedule (ADOS) [29]. Intelligence was measured by the Wechsler Abbreviated Scale of Intelligence test (WASI) [30]. All participants completed baseline self-reported questionnaires of autistic traits (Autism-Spectrum Quotient) [31], obsessionality (Obsessive–Compulsive Inventory-Revised) [32] and current symptoms of ADHD (Barkley Adult ADHD Rating Scale-IV) [33]. Symptoms of anxiety and depression were assessed using The Hamilton Rating Scales for Depression [34] and Anxiety [35]. All participants gave written, informed consent after receiving a complete description of the study. The study had National Research Ethics approval following review by the Stanmore Ethics Committee, London, UK.

### Tianeptine administration procedure

Participants were required to complete two scanning sessions: one after receiving a single dose of 12.5 mg of encapsulated tianeptine and one after receiving a dose of encapsulated placebo (ascorbic acid), in a randomized, double-blind, crossover design. A list of blinding numbers were produced independently and passed directly to the pharmacy in the outpatient department of the Maudsley Hospital, South London & Maudsley NHS Trust, London, UK, using a computerized random number generator with blocked randomization. The pharmacy used these numbers to blind each dose (placebo; tianeptine) as they were encapsulated. Both subject and researcher(s) were blind to dosing throughout data acquisition. The randomization and encapsulation were conducted according to Good Medical Practice and in accordance with CONSORT and SPIRIT guidelines. Each dose was given to the participant 1 h prior to scanning, as tianeptine reaches its peak plasma level after approximately 1 h [36]. There was a minimum of eight days between the scans to allow for complete washout of the drug (t½ = 3 h; washout = 5*t½ = 15 h). All participants received a screening by a medical doctor before and after the administration of both doses.

### Visual analogue scale

All participants completed self-report visual analogue scale (VAS) questionnaires prior to drug administration and after the MRI scan. Side effects potentially associated with tianeptine were measured, including palpitations, nausea, dizziness, attentiveness, anxiety and irritability.

### Go/No-Go inhibition fMRI task

In order to probe the brain’s response inhibition system, participants engaged in a Go/No-Go task (GNG) during each scanning session [8, 37]. During this task, participants made either a motor response on a button box to Go signals or inhibited this response to No-Go signals. In this task, arrows appear pointing to either the left or right side of the screen. The participant responds by pressing the left or right button as fast as possible on a diamond-shaped keypad. Infrequently (12%), arrows pointing to the top (No-Go signals) appear. Subjects have to inhibit any motor response to these stimuli. In 12% of trials, slightly slanted (45 degrees) arrows pointing left or right (oddballs) appear and subjects have to respond as fast as they can, in the same way as for Go signals. No-Go responses were compared to successful oddball trials [8, 37]. There are two reasons we used the oddball instead of Go trials for the comparison. Firstly, this was done in order to control for the oddball effect of the No-Go trials. The No-Go trials are different from the Go trials and appear with less frequency, eliciting the so-called oddball attention effect. Participants pay more attention to rare stimuli than to high-frequent stimuli. Hence, the No-Go trials in addition to measuring inhibition also measure attention allocation to oddball stimuli. Furthermore, in order to control for this effect we added the oddball stimuli and contrasted No-Go with these oddball trials. Secondly, the Go trials appear with higher frequency than the No-Go trials. Hence, the oddball trials furthermore allow us to compare the same amount of No-Go vs oddball ('Go') stimuli.

### Sustained attention fMRI task

In order to probe the brain’s sustained attention network system, the Sustained Attention task (SAT) was performed during each scanning session [7, 12, 13]. In this task, participants need to respond via a right-hand button response as quickly as possible (i.e. within 1 s) to the appearance of a visual timer counting up in milliseconds. When they press the button, the counter shows their reaction time in milliseconds. The visual stimuli appear either after short, predictable consecutive delays of 0.5 s (260 stimuli in total), in series of 3–5 consecutive stimuli or after unpredictable time delays of 2, 5 or 8 s (20 each), which are pseudo-randomly interspersed into the blocks of 3–5 delays of 0.5 s. The long, infrequent, unpredictable delays place a higher load on sustained attention, as participants have to wait for them to occur and they do not know the exact time when they will occur (2 s, 5 s or 8 s)—whereas the short, predictable 0.5-s delays appearing in a row are typically anticipated. Participants learn to estimate the 0.5 s and know that there will be several stimuli appearing in a row [38], placing a higher demand on sensorimotor synchronization [12].

We have previously consistently shown with this task that sustained attention networks are activated during the long relative to the short delays with progressively increasing activation in these networks from 2 to 8 s [7, 12, 13]. Here, we only report on the longest delay that elicits the strongest sustained attention activation, i.e. 8-s vs 0.5-s delays.

### Baseline characteristics and task performance statistical analyses

Statistical tests were performed using the SPSS software (v23.0) [39]. *T*-tests were used to compare baseline characteristics between groups and multivariate analysis of variance (MANOVA) determined any differences in performance and visual analogue scale outcome measures between group and drug conditions. Analysis of variance (ANOVA) was used to compare the largest displacement in head movement between group and drug conditions.

For the GNG task, the performance measures included: probability of inhibition (main inhibitory measure), mean reaction time to the Go signal (motor execution measure) and mean reaction time to the oddball signal. For the SAT task, the performance measures included: coefficient of variation (variation in reaction time during performance of the task adjusted for reaction time, i.e. standard deviation of reaction time divided by reaction time), mean reaction time, premature responses and omission errors.

### fMRI image acquisition

All participants were scanned at the Centre for Neuroimaging Sciences, King’s College London, on a 3-T General Electric Signa HD × Twinspeed scanner (Milwaukee, Wisc.), fitted with a quadrature birdcage head coil. For the fMRI, we acquired T2*-weighted volumes (GNG = 260; SAT = 480) on non-adjacent slices (GNG = 37;SAT = 31) parallel to the anterior–posterior commissure. For GNG, imaging parameters were: TE = 30 ms, TR = 1.8 s, flip angle = 73°, slice thickness = 3.0 mm, in-plane voxel size = 3.75 mm^2^, slice gap = 0.7 mm and matrix size = 64 × 64 voxels. For SAT they were: TE = 30 ms, TR = 1.5 s, flip angle = 68°, slice thickness = 3.0 mm, in-plane voxel size = 3.75 mm^2^, slice gap = 1.4 mm, and matrix size = 64 × 64 voxels.

Also, a high-resolution gradient echo structural scan was sagittally acquired to be used during normalization of the fMRI data into Talairach space. Imaging parameters were: TE = 30 ms, TR = 3 s, flip angle = 90°, 43 slices, slice gap = 0.3 mm, slice thickness = 3.0 mm, matrix size = 128 × 128 voxels.

### fMRI image analysis

The fMRI data were analysed using the XBAM (version 4) software developed at the King’s College London’s Institute of Psychiatry, Psychology and Neuroscience [40]. The associated methods are described in brief in this section and in more detail in Additional file 1. This nonparametric approach minimizes assumptions involved in image processing and has been previously described [26]. Within each run, every volume was realigned to the mean of all the images in the run and then smoothed (in native space) using a Gaussian filter (full-width at half-maximum 8.8 mm). Using a wavelet-based resampling method, a time-series analysis was conducted on each individual subject, in order to compute a sum of squares (SSQ) ratio reflecting the BOLD effect. SSQ ratio maps were transformed into standard stereotactic space [41] using a two-stage warping procedure [40]. First, an average image intensity map for each individual was computed and then warped onto their structural scan. A second stage process then transformed each of these maps from structural space to Talairach space by maximizing the correlation between the images at each stage. The SSQ ratio maps were then transformed into Talairach space using these same two transformations. Group brain activation maps were computed for each drug condition with hypothesis testing performed at both the voxel and the cluster level. Using data-driven, permutation-based methods, with minimal distributional assumptions, time-series analyses were performed for group maps and inter-group random permutation for within-/between-group ANOVAs to compute the distribution of the SSQ ratio under the relevant null distribution hypothesis. Thresholding to the required level of significance was then performed using a two-stage process: first at a voxel-wise *p*-value of 0.05, followed by grouping the supra-threshold voxels into 3D clusters and testing their significance against a null distribution of clusters occurring by chance in the permuted data. The cluster-wise *p-value* can thus be set in such a way as to yield less than one false-positive 3D cluster per map. For GNG, brain activations during No-Go responses were compared to brain activations during successful oddball trials. For SAT, brain activations during 8-s delays were compared to brain activations during 0.5-s delays. A group brain activation map was produced for each group (TD, ASD) and medication (placebo, tianeptine) status. Finally, all ANOVA analyses were conducted with voxel level *p* < 0.05 and a cluster level *p* < 0.02 determined as described above.

### Between-group analysis of variance

A main effect of group (ASD, TD) analysis was conducted for the placebo condition for both GNG and SAT.

To investigate whether brain activation differences in the ASD group relative to the control group under placebo changed after tianeptine dose in ASD, a main effect of group analysis was conducted in regions showing a main effect of group under placebo, but now comparing the control group on placebo with the ASD group on tianeptine, to test whether tianeptine would abolish the baseline differences.

Furthermore, a within ASD effect of drug analysis was conducted, in regions showing a main effect of group, to investigate whether the degree of change in activation in ASD following tianeptine was significant.

### Group x drug status interaction analysis of variance

A two-group (ASD, TD) by two-drug status (placebo, tianeptine) factorial repeated-measures ANOVA was conducted for each task. This analysis investigates how the BOLD response changes in brain regions in each group depending on drug status. The cluster-level threshold was adjusted to *p* < 0.02, resulting in less than one false-positive cluster per map.

### Correlations between symptomatology and change in functional activations

Pearson’s correlations were conducted in XBAM to investigate any associations between core symptoms (as measured by the ADI-R and ADOS, 5 symptoms in total) and differences in BOLD response between tianeptine and placebo conditions (tianeptine–placebo) within ASD, in regions showing a main effect of group during placebo, during both tasks (8 regions in total). The SSQ ratio was extracted for each cluster showing a correlation and plotted versus symptomatology. A false-discovery-rate analysis was conducted to account and correct for multiple comparisons (5 * 8 = 40 comparisons in total).

## Results

### Baseline characteristics

The groups did not significantly differ in age and IQ. As expected, control subjects scored significantly lower on baseline autistic traits and symptoms of anxiety, obsessionality, depression, inattention (childhood) and hyperactivity (currently and in childhood). There was no significant difference between groups in current inattention scores (see Table 1).

**Table 1:** Subjects characteristics

	ASD (*n* = 19)	TD (*n* = 19)	*t*-test *p* value
Age	30 ± 11 (19–50)	27 ± 9 (19–52)	0.3
IQ	113 ± 14 (79–139)	115 ± 10 (88–130)	0.7
ADI-R—Communication	17 ± 9	–	
ADI-R—Social Interaction	14 ± 8	–	
ADI-R—Repetitive Behaviour	5 ± 2	–	
ADOS—Communication	3 ± 2	–	
ADOS—Social Interaction	6 ± 2	–	
AQ	31 ± 11	12 ± 7	< 0.001***
HAM-D	6 ± 4	2 ± 3	0.001**
HAM-A	8 ± 6	3 ± 4	0.003**
OCI-R	23 ± 13	8 ± 9	< 0.001***
GAD-7	7 ± 5	3 ± 3	0.01*
Barkley Inattention Childhood Self	3.3 ± 3.0	0.7 ± 1.2	0.002**
Barkley Hyperactivity Childhood Self	3.7 ± 2.9	1.1 ± 1.9	0.004**
Barkley Inattention Currently Self	1.4 ± 1.8	0.7 ± 1.5	0.2
Barkley Hyperactivity Currently Self	1.2 ± 1.3	0.4 ± 0.8	0.004**

Data in table are shown as mean ± standard deviation (range) (*n* = number of participants). *n* = 19 for sustained attention task and *n* = 17 for Go/No-Go task, which did not significantly affect between-group differences in baseline characteristics or visual analogue scale measures

*TD* typically developed controls, *ASD* individuals with autism spectrum disorder, *ADOS* Autism Diagnostic Observation Scale, *ADI-R* Autism Diagnostic Interview-Revised, *AQ* Autism Quotient, *HAM-D* Hamilton Depression Rating Scale, *HAM-A* Hamilton Anxiety Rating Scale, *OCI-R* Obsessive–Compulsive Inventory Revised, *GAD-7* Generalized Anxiety Disorder Assessment

Between group *t*-test: *p < 0.05; **p < 0.01; ***p < 0.001

### Visual analogue scales

Despite baseline group differences in associated symptomatology (see Additional file 1: Table 1), multivariate analysis of variance showed no significant difference after placebo or tianeptine intake in both groups on subjective reports of physical and psychological side effect symptoms including palpitations, nausea, dizziness, attentiveness, anxiety, depression and irritability (see Additional file 1: Table 1).

### fMRI task performance

#### Go/No-Go task

Multivariate analysis of variance revealed no significant between-group or within-group differences for the probability of inhibition or mean reaction time to the Go or oddball stimuli (see Additional file 1: Table 2).

**Table 2:** Anatomical location and statistics for BOLD activation

Region	*X*	*Y*	*Z*	Cluster *p* value	Cluster size
*GO/NO-GO task (n = 17 for both groups)*					
*ASD placebo vs TD placebo*					
*ASD* < *TD (blue)*					
Right postcentral cortex	58	− 19	33	0.009	144
*ASD* > *TD (red)*					
Right cerebellum	29	− 67	− 40	0.02	81
Right occipital cortex	11	− 96	− 7	0.01	114
Left inferior frontal cortex/left insula	− 40	19	13	0.02	95
Right premotor cortex	43	− 7	50	0.01	111
*ASD tianeptine vs TD placebo*					
*ASD < TD (blue)*					
Right lingual cortex	11	− 100	− 3	0.007	45
*Interaction of drug status (placebo, tianeptine) by group (ASD, TD)*					
Cerebellum bilaterally/limbic area					
Right rostromedial frontal	25	− 19	− 20	0.008	357
Cortex/caudate/cingulate	29	59	7	0.009	376
*Sustained attention task (n = 19 for both groups)*					
*TD placebo vs ASD placebo*					
*ASD* < *TD (blue)*					
Right Middle Temporal Cortex	61	− 7	− 7	0.02	212
Right cuneus	14	− 93	3	0.0008	1082
Left precuneus	− 4	− 63	46	0.009	747
*TD placebo vs ASD tianeptine*					
*ASD* < *TD (blue)*					
Right cuneus	14	− 93	3	0.003	385
*Interaction of drug status (placebo, tianeptine) by group (ASD, TD)*					
Right middle temporal cortex	40	− 56	10	0.01	204
Right thalamus	7	− 11	7	0.001	502
Left middle frontal cortex	− 51	4	46	0.02	180

*X*, *Y*, *Z* = Peak Talairach coordinates

*BOLD* blood-oxygen-level-dependent, *ASD* Individuals with Autism Spectrum Disorder, *TD* Typically Developed Controls

#### Sustained attention task

Multivariate analysis of variance revealed significant differences between ASD and TD during both placebo and tianeptine conditions: slower mean reaction time and higher intrasubject variability for the 0.5- and 8-s delays in ASD compared to controls. More omission errors in ASD compared to controls were observed during the placebo (but not tianeptine) condition for the 0.5-s delay. There were more premature responses in ASD compared to controls during both drug conditions for the 0.5-s delay. There were no significant within-group differences in performance outcome following tianeptine in both groups. When comparing control subjects during placebo with ASD cases during tianeptine for all performance measures, ASD cases performed significantly worse compared to controls for the 0.5-s delay, but there were no significant differences for the 8-s delay (see Additional file 1: Table 3).

### Movement

#### Go/No-Go task

Analysis of variance revealed, for largest head displacement in 3-dimensional space, no significant effect of group (*F*(1, 64) = 1.64; *p* = 0.21), drug (*F*(1, 64) = 1.39; *p* = 0.24) or group x drug interaction (*F*(1, 64) = 0.003; *p* = 0.95) (see Additional file 1: Table 4).

#### Sustained attention task

Analysis of variance revealed, for largest head displacement in 3-dimensional space, no significant effect of group (*F*(3, 72) = 3.13; *p* = 0.08), drug (*F*(3, 72) = 2.00; *p* = 0.16) or group x drug interaction (*F*(3, 72) = 0.22; *p* = 0.64) (see Additional file 1: Table 4).

### Within-group brain activations

#### Go/No-Go task

The group activation maps for each group and drug status revealed significant activation during successful inhibition (No Go > oddball) in inhibitory modulating regions including the inferior, medial, middle frontal and premotor cortex and cerebellum (see Additional file 1: Fig. 1 and Tables 5–8).

#### Sustained attention task

The group activation maps for each group and drug status revealed significant activation during sustained attention (8 s > 0.5 s) in the superior and middle frontal, superior and middle temporal, occipital and pre- and postcentral cortices and cerebellum (see Additional file 1: Fig. 2 and Tables 9–12).

### Between-group differences in brain activation during placebo and tianeptine

#### Go/No-Go task

**Fig. 1:**
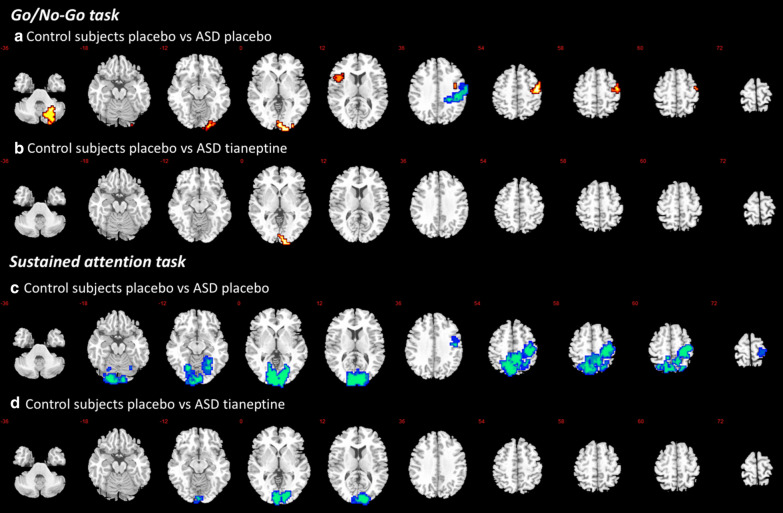
Brain activation map showing abnormally activated regions during response inhibition and sustained attention in ASD that were no longer observed following tianeptine administration; *p* < 0.02 at cluster level. Location of BOLD signal changes between groups. Red: ASD > TD; Blue: ASD < TD. Numeric label = z Talairach coordinate. Right hemisphere of brain is on the right side of the image. *BOLD* blood-oxygen-level-dependent, *ASD* Individuals with autism spectrum disorder, *TD* typically developed controls

During placebo, subjects with ASD relative to TD showed a decrease in BOLD signal in the right postcentral cortex (*p* = 0.009, cluster size = 144 voxels). By contrast, increased activation in ASD compared to TD was observed in the left inferior frontal cortex/left insula (*p* = 0.02, cluster size = 95 voxels), right premotor cortex (*p* = 0.01, cluster size = 111 voxels), right cerebellum (*p* = 0.02, cluster size = 81 voxels) and right occipital cortex (*p* = 0.01, cluster size = 114 voxels) (see Fig. 1a and Table 2).

**Fig. 2:**
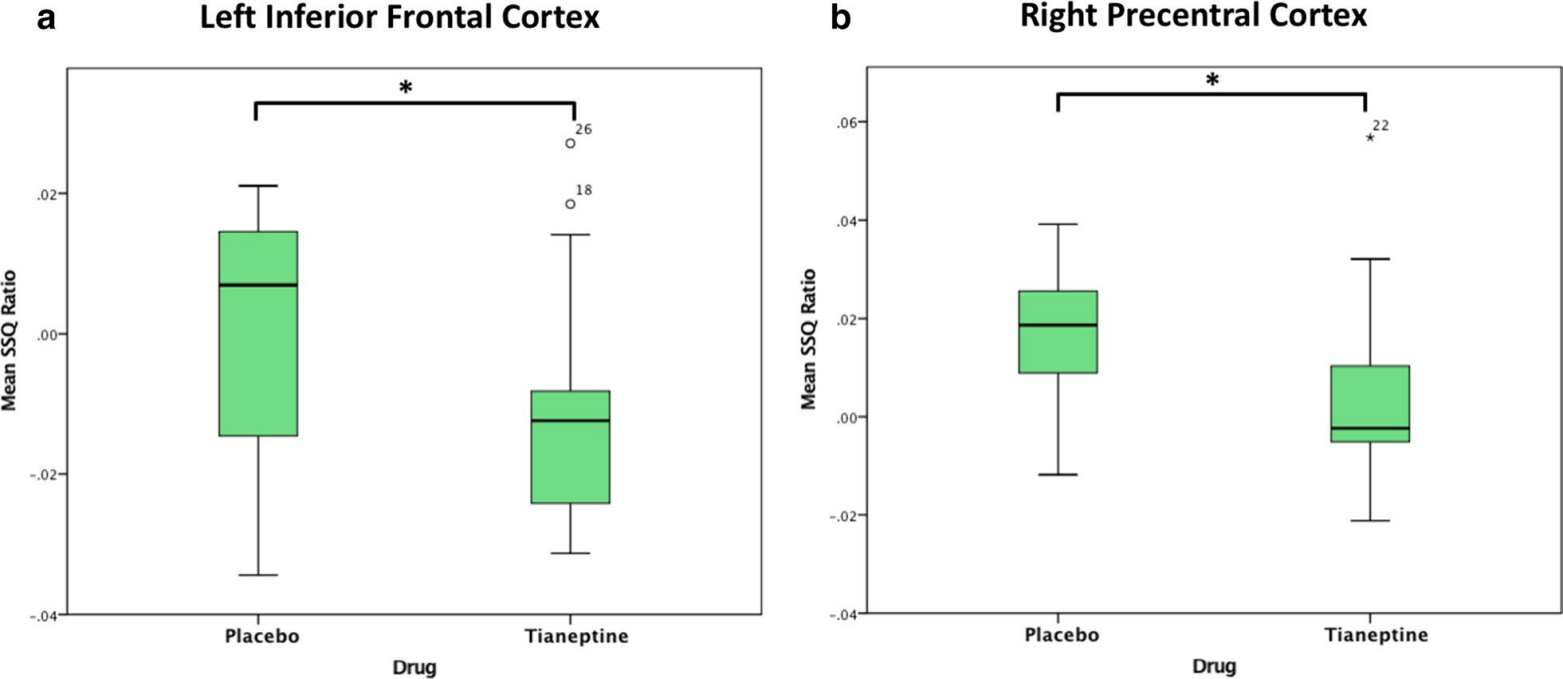
Significant decrease in brain activation during performance of the Go/No-Go task in left inferior frontal and right precentral cortices within ASD following tianeptine administration. *SSQ* sum of squares (statistical measure of BOLD response). *p < 0.05

In order to test whether tianeptine would abolish the baseline differences, subjects with ASD after the tianeptine dose were compared to TD individuals after the placebo dose, focusing on regions where between-group differences under placebo were observed. Nearly all between-group differences were abolished, leaving only one small increase in activation in subjects with ASD compared to controls in the right lingual cortex (*p* = 0.007, cluster size = 45 voxels) (see Fig. 1b and Table 2). Subsequently, a within-ASD analysis was conducted to investigate the effect of drug in those regions specifically. A significant decrease in brain activation was observed in the left insula (*p* = 0.04, see Fig. 2) and right precentral cortex (*p* = 0.01, see Fig. 2).

#### Sustained attention task

During placebo, subjects with ASD relative to TD showed decreased BOLD signal in the right middle temporal cortex (*p* = 0.02, cluster size = 212 voxels), right cuneus (*p* = 0.001, cluster size = 1082 voxels) and left precuneus (*p* = 0.009, cluster size = 747 voxels) (see Fig. 1c, Table 2).

In order to test whether tianeptine would abolish the baseline differences, subjects with ASD during tianeptine were compared to TD during placebo focusing on regions where between-group differences under placebo were observed. Nearly all of these between-group differences were no longer observed, leaving only one small decrease in activation in subjects with ASD compared to TD in the right cuneus (*p* = 0.003, cluster size = 385 voxels) (see Fig. 1d and Table 2). Subsequently, a within-ASD analysis was conducted to investigate the effect of drug in those regions specifically. In none of the regions, brain activation changed significantly.

### Group by drug interaction effects

#### Go/No-Go task

**Fig. 3:**
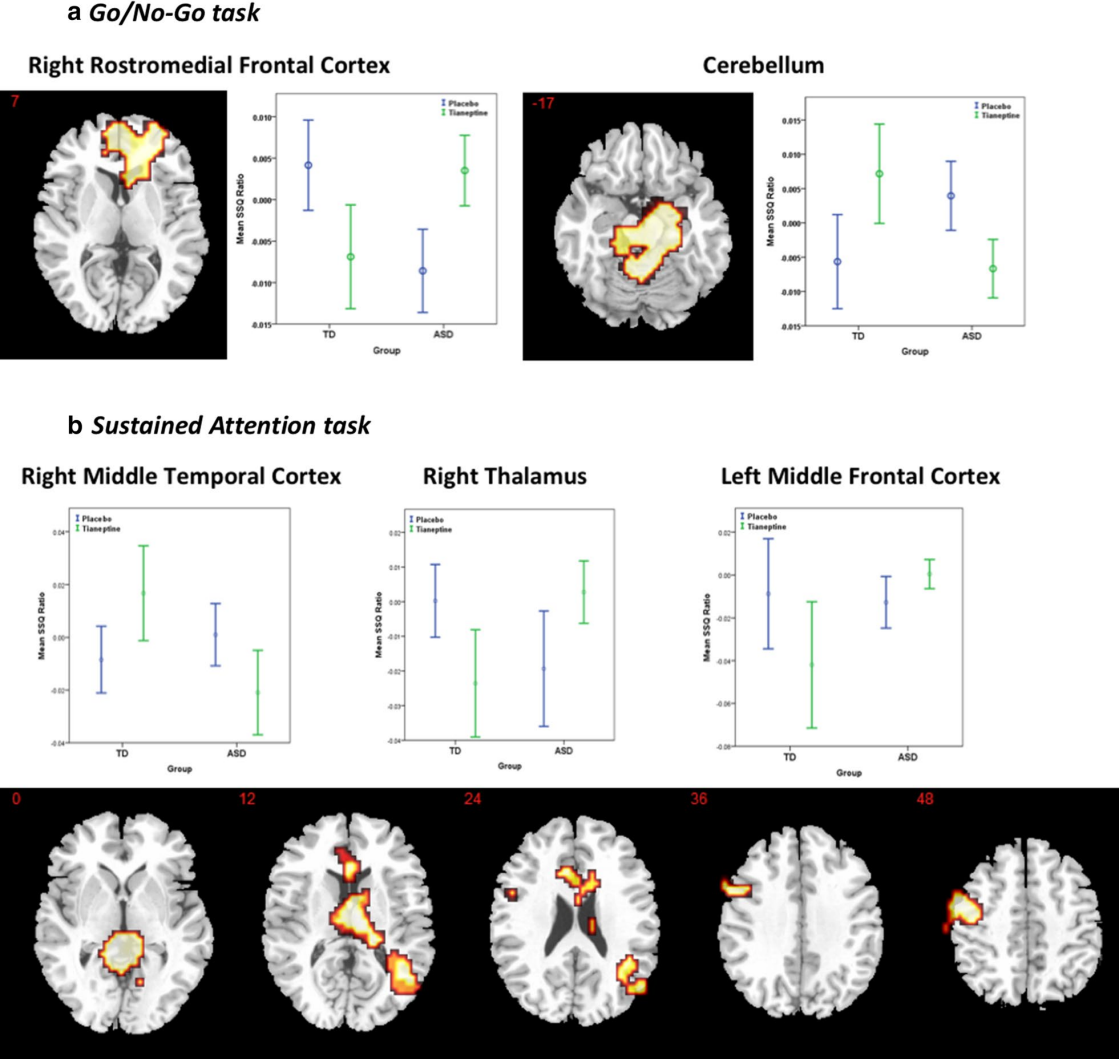
Interaction of drug status (placebo, tianeptine) by group (ASD, TD) during EF; *p* < 0.02 at cluster level. Location of BOLD signal for ANOVA interaction. Numeric label = z Talairach coordinate. Box plots: Mean BOLD signal extracted from each interaction cluster. Right hemisphere of brain is on the right side of the image. *SSQ* sum of squares fMRI statistic, *BOLD* blood-oxygen-level-dependent, *ASD* Individuals with autism spectrum disorder, *TD* Typically developed controls

There were significant interaction effects of BOLD signal response between drug status (placebo, tianeptine) and group (ASD, TD) in two clusters including the right rostromedial frontal cortex (extending into anterior cingulate cortex and caudate; *p* = 0.008, cluster size = 357 voxels) and the cerebellum bilaterally (extending into parahippocampal cortex; *p* = 0.009, cluster size = 376 voxels). In the right rostromedial frontal cortex tianeptine decreased BOLD signal in the TD group, whereas it increased BOLD signal in ASD. The opposite was observed in the cerebellum (see Fig. 3a and Table 2).

#### Sustained attention task

There were significant interaction effects of BOLD signal response between drug status (placebo, tianeptine) and group (ASD, TD) in three clusters. These included the right middle temporal cortex (*p* = 0.01, cluster size = 204 voxels), right thalamus (*p* = 0.001, cluster size = 502 voxels) and left middle frontal cortex (*p* = 0.02, cluster size = 180 voxels). In the right middle temporal cortex tianeptine increased BOLD signal in the TD group, whereas it decreased BOLD signal in ASD. The opposite pattern was observed in the right thalamus and left middle frontal cortex where tianeptine decreased activation in the TD group and increased it in ASD (see Fig. 3b and Table 2).

### Pearson’s correlations between functional activations and symptomatology within ASD

#### Go/No-Go task

Within ASD, we observed correlations between change in brain activation following tianeptine and the severity of RSRBs. The degree of BOLD signal change between tianeptine and placebo correlated positively with severity of RSRBs in the right precentral cortex (*r* = 0.90, *p* =  < 0.001; at baseline ASD < TD, extending from right postcentral cortex) and negatively in the right cerebellum (*r* = −0.74, *p* =  < 0.02, at baseline ASD > TD). Hence, the more severe an individual’s RSRB scores were at baseline, the more likely their BOLD signal in the right precentral cortex and cerebellum would shift towards control levels after tianeptine.

#### Sustained attention task

Within ASD, we observed a correlation between brain activation and communication. Under placebo condition, there was a significant negative correlation between BOLD signal in the right cuneus and ADOS communication scores (*r* = −0.59, *p* = 0.01).

## Discussion

To our knowledge, this is the first study to investigate the effect of tianeptine on brain activation (i.e. BOLD signal change) in adult males with ASD. We observed, under placebo condition, atypical brain activation in ASD during successful completion of the GNG, in the inferior frontal cortex, premotor regions and in the cerebellum. During completion of the SAT, we observed reduced brain activation in ASD compared to TD in the right middle temporal cortex, right cuneus and left precuneus. Furthermore, within the ASD group, the degree of atypical brain activation in the right cuneus was associated with ASD symptom severity (ADOS communication domain). Also, ASD cases performed significantly worse in SAT, as compared to TD. Following tianeptine exposure, within ASD, most brain-functional differences during both tasks were abolished and during response inhibition brain activation in the left inferior frontal and right premotor cortices shifted significantly towards control levels. Further work is required to investigate whether this shift of brain activation during EF tasks is maintained by prolonged tianeptine treatment.

Also, within individuals with ASD we observed correlations between the degree of change in BOLD signal (tianeptine–placebo) during response inhibition in the cerebellum and precentral cortex and the severity of RSRBs. These results are in line with a previous study of adult males with ASD that employed the same scanning paradigm, but decreased brain 5-HT with ATD [9]. This study also reported functional abnormalities in brain activation in the inferior frontal cortex and cerebellum. In addition, increased activation in the left inferior frontal cortex in ASD has previously been reported during motor response inhibition using the same GNG, during cognitive interference inhibition [8] and consistently in a meta-analysis of cognitive control fMRI studies [11]. Taken together, this provides preliminary evidence suggesting that tianeptine affects brain regions associated with RSRBs, though it is unknown if it can successfully treat RSRBs in ASD.

The findings of decreased brain activation during sustained attention in the right middle temporal cortex, right cuneus and left precuneus differ somewhat from previous fMRI studies using the same task in ASD. In these studies decreased brain activation was observed in prefrontal, parietal, temporal, striato-thalamic and cerebellar regions as well as a negative correlation with brain activation and age in the left precuneus and right occipital cortex [7, 12]. The dissimilarity in findings may be explained by the different age ranges studied. One study only included children (ages: 11–17 years) with ASD [12], while the other study sample consisted of a mixture of children and adults (ages: 11–35 years) [7]. Nonetheless, our findings support the suggestion that individuals with ASD have abnormalities in brain activation during sustained attention—although the specific brain regions affected may be age-dependent.

The observed differences in task performance in ASD included slower mean reaction times and larger intrasubject response variability. This is consistent with what has been seen in previous studies in children and adults with ASD during attention tasks [7, 42]. Although tianeptine did not significantly change sustained attention performance in both groups, some performance abnormalities in ASD were no longer significant following tianeptine. This is in line with a study that reported improvement in neurocognitive functions in a neurotypical population after 12 weeks of treatment with tianeptine [25].

Further to the main effects of group, the interaction analysis results showed that, during response inhibition, within the control group, tianeptine decreased brain activation in the rostromedial frontal cortex and caudate, whereas in ASD brain activation was increased. In contrast, within the control group, tianeptine increased brain activation in the cerebellum, whereas it decreased cerebellum activation in ASD. This ‘reversal’ of brain activation may reflect altered functionality of frontal-cerebellar networks. Atypical connectivity of white matter within the cerebellum and its mid-brain and cortical projections have been observed in ASD [43, 44]. Studies investigating functional connectivity support these findings, suggesting abnormalities in connections between the cerebellum and both motor and non-motor cortical regions [45, 46]. Furthermore, the interactions found here are similar to previous reports of the impact of ATD on brain function in ASD compared to controls: the same direction of increases and decreases of brain activation was previously reported in the frontal cortex and cerebellum [9]. For the sustained attention task, the interaction analysis revealed three significant clusters where tianeptine shifted brain activation in opposite directions in ASD compared to controls. Within the control group, tianeptine increased brain activation in the right middle temporal cortex, whereas in ASD brain activation was decreased. In contrast, within the control group, tianeptine decreased brain activation in the right thalamus and left middle frontal cortex, whereas it increased brain activation in ASD. All these regions have previously been reported to either show abnormal brain activation and/or functional maturation in ASD during sustained attention [7]. In addition, our results are in line with prior imaging studies in ASD [47, 48]. This suggests that brain regions implicated in the abnormal neurodevelopmental trajectory of ASD continue to show differences in adulthood, and this may have implications for treatment response. Taken together, our work and that of others suggest that the neuropharmacological mechanism underpinning response to tianeptine is different in ASD as compared to controls; therefore, treatments commonly used in neurotypical populations may not be as ‘translatable’ to individuals with ASD as currently assumed. Moreover, recently published treatment guidelines in ASD reported that evidence for the effectiveness of selective serotonin reuptake inhibitors (SSRIs) is limited [49]. Thus, pharmacological interventions affecting the serotonergic system need to be tested specifically in ASD as similar results to those found in neurotypical populations cannot be assumed.

Whether the impact of both ATD and tianeptine on abnormal brain activation in ASD is explained by the same neurochemical pathway—the serotonergic system—is unknown. Tianeptine was initially considered to be a selective serotonin reuptake enhancer (SSRE) as its acute and long-term administration decreased extracellular 5-HT levels in the brain stem, striatum, cerebral cortex and hippocampus in rats [22, 23]. Later studies, however, contradicted these findings—albeit this discrepancy may be explained by technical differences in the micro-dialysis techniques employed. Nevertheless, recent evidence in humans demonstrates a reduction in plasma serotonin and increase in platelet serotonin following acute administration of tianeptine, consistent with the effect of enhanced serotonin reuptake [50]. In addition, tianeptine has also been shown to: (1) regulate stress-induced glutamate release, (2) modulate plasticity in the amygdala; (3) reverse stress-induced hippocampal dystrophy [51]; and (4) be a *μ*-opioid receptor agonist [52]. Given that we only investigated the brain response after a single dose of tianeptine, it is unlikely that our results are due to modulating plasticity or the reversal of dystrophy. However, we cannot rule out the possibility that our results may be partially explained by modulation of the glutamatergic and μ-opioid systems. For example, the glutamatergic system has been widely reported to be abnormal in ASD [53]. Also, alterations in the μ-opioid system have been proposed to contribute to ASD [54]. Hence, future studies are required to investigate which of the proposed mechanisms underlie the reported modulating effect.

### Limitations

We conducted a pilot study using a single dose of tianeptine and so we cannot comment on long-term outcome. Nevertheless, this study does provide a first necessary proof of concept for a potential treatment targeting ASD symptomatology. Also, a potential problem with fMRI adaptations of response inhibition tasks is that motor responses to Go trials are compared to non-motor responses to No-Go trials. Thus, some activation differences could have been potentially motor-related rather than purely inhibition-related. Last, we did not find differences in performance outcome between groups for the GNG. A recent study using a much larger sample (201 ASD cases and 240 controls) employed online GNG and reported deficits in response inhibition that were associated with diagnosis and autistic traits [6]. In contrast, lack of performance difference was reported in a similarly sized fMRI study using the GNG [9]. Hence, our sample size may be underpowered to detect behavioural data differences. However, the sample size was large enough to detect brain activation differences, which have previously been reported to be more sensitive to drug effects than behaviour, including in fMRI studies of ASD [9, 55].

## Conclusions

We report that tianeptine can abolish most case–control differences in brain function during EF tasks; within ASD it can significantly shift brain activation deficits associated with RSRB towards control levels. This suggests a potential utility of tianeptine for targeting core or associated symptoms in ASD. Hence, future trials should investigate whether the shift in brain activation we discovered following a single dosage of tianeptine is maintained after prolonged treatment, and whether this is associated with response to treatment.

## Supplementary Information

**Additional file 1.** Supplementary material.

## Data Availability

The dataset used and/or analysed during the current study are available from the corresponding author on reasonable request.

## Abbreviations

ASD: Autism spectrum disorder
EF: Executive functioning
RSRBs: Restricted, stereotyped and repetitive behaviours
ADHD: Attention deficit hyperactivity disorder
fMRI: Functional MRI
SSRI: Selective serotonin reuptake inhibitor
ATD: Acute tryptophan depletion
TD: Typically developed control participants
ADI-R: Autism Diagnostic Interview–Revised
ADOS: Autism Diagnostic Observation Schedule
WASI: Wechsler Abbreviated Scale of Intelligence test
VAS: Visual analogue scale
GNG: Go/No-Go task
SAT: Sustained attention task
MANOVA: Multivariate analysis of variance
ANOVA: Analysis of variance
SSQ: Sum of squares
SSRE: Selective serotonin reuptake enhancer
