# Supplementary material for: Modulation of atypical brain activation during executive functioning in autism: a pharmacological MRI study of tianeptine
Source: Mol Autism. 2021 Feb 19;12:14. doi: 10.1186/s13229-021-00422-0 (PMC7893772; doi:10.1186/s13229-021-00422-0)
Supplement: Supplementary file 1 — Additional file 1. Supplementary material. [file 13229_2021_422_MOESM1_ESM.docx]

## Supplementary material

## fMRI data analysis XBAM

Data were analyzed with software developed at the Institute of Psychiatry, London, (XBAM) using a non-parametric approach (for a full description and references, see **www.brainmap.co.uk**). XBAM uses median statistics to control for outlier effects and employs permutation rather than normal theory based inference. Furthermore, its most common test statistic (sum of squares ratio [SSQ]) is computed by standardising for individual difference in residual noise before embarking on second level, multi-subject testing using robust permutation-based methods. This allows a mixed effects approach to analysis to deal with the issue of non-normality. The use of a mixed effect approach addresses the issue of inequality of individual residual variances by effectively “down weighting” responses with large residual variances. The significance of the resulting reweighted responses at group level is then tested by data permutation to avoid assumptions of normality.

## Individual brain activation mapping

Within each run, the T2-weighted signal intensity time series at each voxel of the realigned images were regressed on the concomitant and lagged time series of estimated positional displacements (δx, δy, δz) at each voxel in order to remove subject-induced motion artifacts (1). For framewise displacement the time-series were also globally detrended at the same time. Slice-timing correction was used as a default-setting, and the data were spatially smoothed using a Gaussian filter (FWHM 8.8 mm) to improve the signal to noise characteristics of the images. Furthermore, for the block design we, by default, used piecewise linear baseline correction while, for event-related, we used a high-pass filter.

Responses to the experimental paradigms were then detected by first convolving each component of the experimental design with each of two gamma variate functions (peak responses at 4 and 8 sec respectively). These two functions were chosen to encompass the known range of times to peak response following stimulus onset for BOLD effects. The best fit between the weighted sum of these convolutions and the time series at each voxel was computed using the constrained BOLD effect model.(2) Following computation of the model fit, a goodness of fit statistic was computed. This consisted of the ratio of the sum of squares of deviations from the mean image intensity (over the whole time series) due to the model to the sum of squares of deviations due to the residuals (SSQ ratio). Following computation of the observed SSQ ratio at each voxel, the data are permuted (50 permutations at each voxel and then all the permutations compiled together to form a big probability distribution) using a wavelet-based method.(3) Repeated application of this strategy at each voxel followed by recomputation of the SSQ ratio from the permuted data allows (by combination of results over all intracerebral voxels) the data-driven calculation of the null distribution of SSQ ratios under the assumption of no experimentally determined response. Using this distribution it is possible to calculate the critical value of SSQ ratio needed to threshold the maps at any desired type I error rate. Then, detection of activated voxels was extended from voxel to cluster level. (4)

## Group brain activation mapping

The observed and permuted SSQ ratio maps for each individual were transformed into the standard space (5) using a two stage warping procedure.(6) For both stages of the warping process, a twelve parameter affine transform was employed. This involves first computing the average image intensity map for each individual over the course of the experiment. The transformations required to map this image to the structural scan for each individual and then from “structural space” to the Talairach template were then computed by maximizing the correlation between the images at each stage. The SSQ ratio maps were then transformed into Talairach space using these transformations. Group activation maps were then computed by determining the median SSQ ratio at each voxel (over all individuals) in the observed and permuted data maps (medians are used to minimize outlier effects). The distribution of median SSQ ratios over all intracerebral voxels from the permuted data was then used to derive the null distribution of SSQ ratios and which can then be thresholded to produce group activation maps at any desired voxel or cluster-level type I error rate. In this two level clustering procedure,(4) the first (voxel-wise) thresholding is carried out at an uncorrected p value of 0.05 to give the maximum allowable sensitivity. In order to eliminate the resulting false positive activations, a second, cluster-level thresholding step is carried out and the threshold of this second step is adjusted to give an expectation of less than one false positive cluster over the whole brain. As the cluster level threshold is set at the whole brain level, the normal, voxel-wise issue of multiple comparisons does not apply. The computation of a standardized measure of effect SSQ ratio at the individual level, followed by analysis of the median SSQ ratio maps over all individuals treats intra- and inter-subject variations in effect separately. This constitutes a mixed-effect approach, which allows for inferences from these results to be made about the larger population.

## Supplementary table 1: Visual analogue scales (VAS) of self-reported symptoms potentially associated with tianeptine

| **Group** | **Drug** | **Time** | **Palpitations** | **Nausea** | **Dizziness** | **Inattentiveness** | **Anxiety** | **Depression** | **Irritability** |
| --- | --- | --- | --- | --- | --- | --- | --- | --- | --- |
| **TD** |  |  |  |  |  |  |  |  |  |
|  | **Placebo** | Baseline | 10.0 ± 16.4 | 9.8 ± 19.2 | 7.1 ± 17.4 | 24.8 ± 25.1 | 8.6 ± 12.7 | 22.1 ± 19.7 | 8.4 ± 17.1 |
|  |  | 2.5 h post drug intake | 3.5 ± 4.0 | 2.7 ± 3.4 | 3.1 ± 4.4 | 26.5 ± 25.6 | 6.5 ± 5.3 | 17.9 ± 16.0 | 4.7 ± 7.4 |
|  |  | *p*-value | 0.1 | 0.1 | 0.3 | 0.8 | 0.5 | 0.5 | 0.4 |
|  |  | *F* (1, 36) | 2.8 | 2.5 | 0.9 | 0.04 | 0.4 | 0.5 | 0.7 |
|  | **Tianeptine** |  |  |  |  |  |  |  |  |
|  |  | Baseline | 5.2 ± 7.4 | 4.9 ± 6.5 | 5.9 ± 9.6 | 32.3 ± 30.2 | 11.6 ± 21.8 | 25.8 ± 25.6 | 5.6 ± 10.3 |
|  |  | 2.5 h post drug intake | 5.3 ± 7.0 | 4.1 ± 5.0 | 3.8 ± 5.1 | 24.1 ± 20.4 | 8.4 ± 12.9 | 18.8 ± 18.7 | 4.0 ± 5.3 |
|  |  | *p*-value | 1.0 | 0.7 | 0.4 | 0.3 | 0.6 | 0.3 | 0.5 |
|  |  | *F* (1, 36) | 0.002 | 0.2 | 0.7 | 1.0 | 0.3 | 0.9 | 0.4 |
| **ASD** |  |  |  |  |  |  |  |  |  |
|  | **Placebo** | Baseline | 3.9 ± 6.3 | 4.7 ± 6.7 | 5.9 ± 9.6 | 33.5 ± 26.5 | 15.7 ± 14.2 | 26.9 ± 23.4 | 8.0 ± 14.3 |
|  |  | 2.5 h post drug intake | 6.5 ± 11.0 | 4.8 ± 8.3 | 5.3 ± 8.2 | 26.9 ± 24.0 | 12.7 ± 17.1 | 24.8 ± 25.2 | 8.3 ± 14.9 |
|  |  | *p*-value | 0.4 | 1.0 | 0.8 | 0.4 | 0.6 | 0.8 | 0.9 |
|  |  | *F* (1, 35) | 0.8 | 0.002 | 0.1 | 0.6 | 0.3 | 0.1 | 0.004 |
|  |  |  |  |  |  |  |  |  |  |
|  | **Tianeptine** | Baseline | 7.4 ± 14.0 | 7.4 ± 12.3 | 8.8 ± 13.7 | 27.1 ± 26.6 | 15.5 ± 18.8 | 31.6 ± 24.2 | 7.1 ± 12.3 |
|  |  | 2.5 h post drug intake | 8.7 ± 13.9 | 9.5 ± 14.0 | 7.2 ± 7.4 | 29.5 ± 20.5 | 14.1 ± 16.2 | 27.1 ± 25.0 | 11.3 ± 15.0 |
|  |  | *p*-value | 0.8 | 0.6 | 0.7 | 0.9 | 0.8 | 0.6 | 0.4 |
|  |  | *F* (1, 36) | 0.1 | 0.3 | 0.2 | 0.03 | 0.1 | 0.3 | 0.9 |

Outcome is shown in mm (between 0 mm (not at all severe) and 100 mm (extremely severe)). Data in table is shown as mean ± standard deviation. Abbreviations: TD, typically developed controls; ASD, individuals with autism spectrum disorder.

## Supplementary table 2: Go/No-Go task performance measures

|  | **TD** |  |  |  | **ASD** |  |  |  | **Difference TD, ASD** | | | |
| --- | --- | --- | --- | --- | --- | --- | --- | --- | --- | --- | --- | --- |
| **Performance measures** | **Placebo** | **Tianeptine** | ***p*-value** | ***F* (1, 31)** | **Placebo** | **Tianeptine** | ***p*-value** | ***F* (1, 32)** | **Placebo  *p*-value** | ***F* (1, 32)** | **Tianeptine *p*-value** | ***F* (1, 31)** |
| P(I) % | 97 ± 3.9 | 95 ± 4.3 | 0.8 | 0.04 | 97 ± 1.8 | 95 ± 4.6 | 0.9 | 0.004 | 0.2 | 1.4 | 0.1 | 2.3 |
| MRT Go (ms) | 463 ± 86 | 463 ± 53 | 0.8 | 0.1 | 456 ± 63 | 466 ± 61 | 0.9 | 0.02 | 1.0 | 0.001 | 0.6 | 0.2 |
| MRT Odd (ms) | 488 ± 96 | 480 ± 51 | 0.8 | 0.1 | 481 ± 75 | 496 ± 68 | 0.4 | 0.6 | 0.8 | 0.1 | 0.5 | 0.4 |

Data in table is shown as mean ± standard deviation. P(I): probability of inhibition; MRT Go: mean reaction time to Go signal; MRT Odd: mean reaction time to Oddball signal. TD: typically developed controls; ASD: individuals with autism spectrum disorder.

## Supplementary table 3: Performance measures sustained attention task

| **Performance Measure** | **Delay (s)** | **TD** | | |  | **ASD** | | |  | **Difference TD, ASD** | | | | **TD placebo vs ASD tianeptine** | |
| --- | --- | --- | --- | --- | --- | --- | --- | --- | --- | --- | --- | --- | --- | --- | --- |
|  |  | **Placebo** | **Tianeptine** | ***p* - value** | ***F***  **(1, 36)** | **Placebo** | **Tianeptine** | ***p* - value** | ***F***  **(1, 36)** | **Placebo**  ***p* - value** | ***F***  **(1, 36)** | **Tianeptine**  ***p* - value** | ***F***  **(1, 36)** | ***p-*value** | ***F***  **(1, 36)** |
| **Mean reaction time (RT) (ms)** | 0.5s  8s | 310 ± 32  410 ± 50 | 310 ± 23  400 ± 44 | 0.9  0.4 | 0.03  0.8 | 350 ± 55  450 ± 50 | 370 ± 66  450 ± 70 | 0.3  0.9 | 0.9  0.02 | 0.03*  0.03* | 5.4  5.2 | 0.002**  0.01* | 11.6  7.6 | 0.004**  0.06 | 9.9  3.9 |
| **Intrasubject variability (SD) of RT (ms)** | 0.5s  8s | 61 ± 23  59 ± 37 | 62 ± 39  51 ± 28 | 0.9  0.4 | 0.01  0.6 | 82 ± 26  85 ± 35 | 90 ± 39  75 ± 48 | 0.5  0.5 | 0.5  0.6 | 0.01*  0.04* | 7.1  4.8 | 0.03*  0.07 | 4.9  3.4 | 0.01*  0.28 | 7.9  1.2 |
| **Omission errors** | 0.5s  8s | 1.6 ± 4.8  0.7 ± 1.9 | 5.9 ± 23  0.4 ± 1.6 | 0.4  0.6 | 0.6  0.2 | 9.4 ± 13  1.1 ± 2.5 | 16 ± 21  1.6 ± 2.2 | 0.3  0.5 | 1.3  0.5 | 0.02*  0.56 | 5.7  0.3 | 0.17  0.06 | 2.0  3.7 | 0.01*  0.17 | 8.1  2.0 |
| **Premature responses** | 0.5s  8s | 0.9 ± 2.0  3.1 ± 3.6 | 0.7 ± 1.3  3.4 ± 4.1 | 0.6  0.8 | 0.2  0.04 | 3.3 ± 4.0  3.4 ± 3.6 | 3.2 ± 3.9  4.1 ± 4.9 | 0.9  0.6 | 0.02  0.2 | 0.03*  0.82 | 5.4  0.1 | 0.01*  0.64 | 7.0  0.2 | 0.04*  0.50 | 4.9  0.5 |

Data in table is shown as mean ± standard deviation. Multivariate ANOVA *=*p*<0.05; **= *p*<0.01; Abbreviations: s, seconds; ms, milliseconds; SD, standard deviation; TD, typically developed controls; ASD, individuals with autism spectrum disorder; RT, mean reaction time

## Supplementary table 4: Subject Movement

| D statistic | TD placebo | TD tianeptine | ASD placebo | ASD tianeptine |
| --- | --- | --- | --- | --- |
| Go/No-Go Task | 1.6 ± 0.6 | 1.8 ± 1.0 | 1.8 ± 0.8 | 2.1 ± 1.0 |
| Sustained Attention Task | 2.5 ± 2.2 | 2.1 ± 1.1 | 3.5 ± 2.6 | 2.7 ± 1.2 |

Data in table is shown as mean ± standard deviation. Abbreviations: TD, Typically Developed Controls; ASD, Individuals with Autism Spectrum Disorder. D statistic: D = SQRT ((Largest_displacement_x*Largest_displacement_x) + (Largest_displacement_y*Largest_displacement_y) + (Largest_displacement_z*Largest_displacement_z)); Largest displacement in mm in three dimensions (x, y and z)

## Supplementary table 5: Anatomical location and statistics for BOLD activation for the Go/No-Go (TD group, placebo condition)

| **Region** | **X** | **Y** | **Z** | **Cluster *p*-value** | **Cluster size** |
| --- | --- | --- | --- | --- | --- |
| **No-Go < Oddball (blue)** |  | | | | |
| Right Cerebellum | 18 | -67 | -40 | 0.03 | 59 |
| Right Putamen | 22 | 4 | 17 | 0.02 | 59 |
| Right Posterior Cingulate | 7 | -56 | 17 | 0.02 | 107 |
| Right Cuneus | 7 | -85 | 30 | 0.006 | 204 |
| Right Medial Frontal Cortex | 29 | -4 | 36 | 0.05 | 29 |
| Left Posterior Cingulate | -22 | -63 | 10 | 0.02 | 106 |
| Left Inferior Frontal Cortex | -40 | 15 | 13 | 0.03 | 78 |
| Left Superior Frontal Cortex | -11 | 44 | 36 | 0.05 | 25 |
| Left Precentral Cortex | -36 | -22 | 53 | 0.004 | 227 |
| **No-Go > Oddball (red)** |  | | | | |
| Right Occipital Cortex | 11 | -93 | 17 | 0.04 | 35 |
| Right Superior Temporal Cortex | 58 | -52 | 20 | 0.04 | 40 |
| Right Precentral Cortex | 61 | 0 | 26 | 0.04 | 34 |
| Right Postcentral Cortex | 43 | -22 | 33 | 0.03 | 67 |
| Left Cerebellum | -29 | -74 | -20 | 0.04 | 41 |
| Left Middle Temporal Cortex | -47 | -59 | 17 | 0.01 | 125 |
| Left Precuneus | 0 | -44 | 50 | 0.04 | 32 |

x, y, z = Peak Talairach coordinates. Abbreviations: BOLD, blood-oxygen-level dependent; TD, typically developed controls; ASD, individuals with autism spectrum disorder.

## Supplementary table 6: Anatomical location and statistics for BOLD activation for the Go/No-Go (TD group, tianeptine condition)

| **Region** | **X** | **Y** | **Z** | | **Cluster *p*-value** | **Cluster size** |
| --- | --- | --- | --- | --- | --- | --- |
| **No-Go < Oddball (blue)** |  | | |  | | |
| Left Middle Temporal Cortex | -36 | -78 | 20 | | 0.01 | 136 |
| Left Middle Frontal Cortex | -43 | 30 | 30 | | 0.01 | 116 |
| Left Superior Frontal Cortex | -22 | 33 | 43 | | 0.01 | 152 |
| Left Postcentral Cortex | -40 | -26 | 43 | | 0.02 | 82 |
| **No-Go > Oddball (red)** |  | | | | | |
| Right Cerebellum | 29 | -63 | -17 | | 0.04 | 30 |
| Right Middle Temporal Cortex | 51 | -48 | -7 | | 0.05 | 43 |
| Right Middle Temporal Cortex | 58 | -30 | 0 | | 0.03 | 50 |
| Right Superior Temporal Cortex | 58 | -37 | 17 | | 0.04 | 45 |
| Right Inferior Frontal Cortex | 47 | 33 | 7 | | 0.009 | 263 |
| Right Inferior Parietal Cortex | 43 | -48 | 46 | | 0.01 | 204 |
| Right Superior Frontal Cortex | 18 | 11 | 53 | | 0.009 | 283 |
| Left Cerebellum | -18 | -78 | -20 | | 0.04 | 30 |

x, y, z = Peak Talairach coordinates. Abbreviations: BOLD, blood-oxygen-level dependent; TD, typically developed controls; ASD, individuals with autism spectrum disorder.

## Supplementary table 7: Anatomical location and statistics for BOLD activation for the Go/No-Go (ASD group, placebo condition)

| **Region** | **X** | **Y** | **Z** | **Cluster *p*-value** | **Cluster size** |
| --- | --- | --- | --- | --- | --- |
| **No-Go < Oddball (blue)** |  | | | | |
| Right Cuneus | 11 | -59 | 7 | 0.01 | 113 |
| Right Superior Frontal Cortex | 22 | 41 | 23 | 0.02 | 97 |
| Left Middle Temporal Cortex | -36 | -59 | 7 | 0.03 | 50 |
| Left Medial Frontal Cortex | -11 | 56 | 10 | 0.02 | 94 |
| Left Postcentral Cortex | -36 | -30 | 50 | 0.008 | 197 |
| **No-Go > Oddball (red)** |  | | | | |
| Right Occipital Cortex | 33 | -89 | -17 | 0.02 | 81 |
| Right Precentral Cortex | 47 | -11 | 50 | 0.01 | 129 |
| Right Medial Frontal Cortex | 4 | 7 | 46 | 0.01 | 117 |
| Left Occipital Cortex | -18 | -93 | -17 | 0.01 | 139 |
| Left Middle Temporal Cortex | -58 | -41 | 17 | 0.05 | 38 |
| Left Precuneus | -22 | -78 | 40 | 0.02 | 58 |
| Left Middle Frontal Cortex | -36 | 19 | 46 | 0.02 | 77 |

x, y, z = Peak Talairach coordinates. Abbreviations: BOLD, blood-oxygen-level dependent; TD, typically developed controls; ASD, individuals with autism spectrum disorder.

## Supplementary table 8: Anatomical location and statistics for BOLD activation for the Go/No-Go (ASD group, tianeptine condition)

| **Region** | **X** | **Y** | **Z** | **Cluster *p*-value** | **Cluster size** |
| --- | --- | --- | --- | --- | --- |
| **No-Go < Oddball (blue)** |  | | | | |
| Left Precentral Cortex | -47 | 11 | 10 | 0.02 | 78 |
| Left Postcentral Cortex | -33 | -33 | 53 | 0.02 | 203 |
| **No-Go > Oddball (red)** |  | | | | |
| Right Occipital Cortex | 11 | -89 | -7 | 0.003 | 348 |
| Right Temporal Cortex | 61 | -48 | -3 | 0.05 | 31 |
| Right Claustrum | 22 | 26 | 0 | 0.03 | 53 |
| Right Middle Frontal Cortex | 51 | 19 | 30 | 0.01 | 75 |
| Right Cuneus | 11 | -81 | 20 | 0.004 | 189 |
| Left Precuneus | -7 | -67 | 43 | 0.04 | 19 |
| Left Medial Frontal Cortex | -11 | 7 | 50 | 0.04 | 36 |

x, y, z = Peak Talairach coordinates. Abbreviations: BOLD, blood-oxygen-level dependent; TD, typically developed controls; ASD, individuals with autism spectrum disorder.

## Supplementary table 9: Anatomical location and statistics for BOLD activation for the sustained attention task (TD group, placebo condition)

| **Region** | **X** | **Y** | **Z** | **Cluster *p*-value** | **Cluster size** |
| --- | --- | --- | --- | --- | --- |
| **8s > 0.5s (red)** |  |  |  |  |  |
| Right Cuneus | 14 | -93 | 3 | 0.0006 | 5629 |
| Left Middle Temporal Cortes | -54 | -33 | 3 | 0.002 | 572 |
| Left Superior Frontal Cortex | -33 | 48 | 33 | 0.002 | 408 |
| **8s < 0.5s (blue)** |  |  |  |  |  |
| Right Cerebellum | 43 | -74 | -17 | 0.0007 | 6123 |
| Bilateral Medial Frontal Cortex | 0 | -4 | 53 | 0.004 | 441 |

x, y, z = Peak Talairach coordinates. Abbreviations: BOLD, blood-oxygen-level dependent; TD, typically developed controls; ASD, individuals with autism spectrum disorder.

## Supplementary table 10: Anatomical location and statistics for BOLD activation for the sustained attention task (TD group, tianeptine condition)

| **Region** | **X** | **Y** | **Z** | **Cluster *p*-value** | **Cluster size** |
| --- | --- | --- | --- | --- | --- |
| **8s > 0.5s (red)** |  |  |  |  |  |
| Right Lingual Cortex | 14 | -83 | -3 | 0.0009 | 6722 |
| Left Middle Frontal Cortex | -25 | 30 | 43 | 0.007 | 344 |
| **8s < 0.5 (blue)** |  |  |  |  |  |
| Right Cerebellum | 33 | -85 | -20 | 0.002 | 3006 |
| Right Middle Temporal Cortex | 33 | -67 | 26 | 0.008 | 398 |
| Left Cerebellum | -32 | -85 | -20 | 0.002 | 1080 |
| Left Postcentral Cortex | -40 | -26 | 50 | 0.002 | 660 |
| Bilateral Medial Frontal Cortex | 0 | -7 | 59 | 0.003 | 525 |

x, y, z = Peak Talairach coordinates. Abbreviations: BOLD, blood-oxygen-level dependent; TD, typically developed controls; ASD, individuals with autism spectrum disorder.

## Supplementary table 11: Anatomical location and statistics for BOLD activation for the sustained attention task (ASD group, placebo condition)

| **Region** | **X** | **Y** | **Z** | **Cluster *p*-value** | | **Cluster size** |
| --- | --- | --- | --- | --- | --- | --- |
| **8s > 0.5s (red)** |  |  |  |  |  | |
| Right Superior Temporal Cortex | 40 | 22 | -30 | 0.004 | 313 | |
| Left Cerebellum | -7 | -78 | -10 | 0.0006 | 4256 | |
| Left Middle Temporal Cortex | -58 | -41 | 0 | 0.006 | 237 | |
| **8s < 0.5 (blue)** |  |  |  |  |  | |
| Right Inferior Frontal Cortex | 58 | 19 | 23 | 0.002 | 412 | |
| Left Postcentral Cortex | -36 | -30 | 56 | 0.0006 | 1148 | |
| Left Cerebellum | -33 | -89 | -20 | 0.0006 | 2656 | |

x, y, z = Peak Talairach coordinates. Abbreviations: BOLD, blood-oxygen-level dependent; TD, typically developed controls; ASD, individuals with autism spectrum disorder.

## Supplementary table 12: Anatomical location and statistics for BOLD activation for the sustained attention task (ASD group, tianeptine condition)

| **Region** | **X** | **Y** | **Z** | **Cluster *p*-value** | | **Cluster size** |
| --- | --- | --- | --- | --- | --- | --- |
| **8s > 0.5s (red)** |  |  |  |  |  | |
| Right Superior Temporal Cortex | 51 | 26 | -26 | 0.01 | 124 | |
| Right Amygdala | 25 | -4 | -17 | 0.009 | 153 | |
| Right Postcentral Cortex | 14 | -41 | 63 | 0.005 | 246 | |
| Left Precentral Cortex | -58 | -4 | 33 | 0.008 | 140 | |
| Left Middle Frontal Cortex | -36 | 37 | 43 | 0.002 | 407 | |
| Bilateral Cuneus | 0 | -74 | 17 | 0.0005 | 1144 | |
| **8s < 0.5 (blue)** |  |  |  |  |  | |
| Right Cerebellum | 40 | -70 | -20 | 0.0006 | 1518 | |
| Left Inferior Occipital Cortex | -29 | -89 | -10 | 0.0006 | 407 | |
| Left Postcentral Cortex | -51 | -26 | 50 | 0.0006 | 513 | |

x, y, z = Peak Talairach coordinates. Abbreviations: BOLD, blood-oxygen-level dependent; TD, typically developed controls; ASD, individuals with autism spectrum disorder.

## Supplementary figure 1: Anatomical location and statistics for BOLD activation per group (TD, ASD) and drug (placebo, tianeptine) condition for the Go/No-Go task


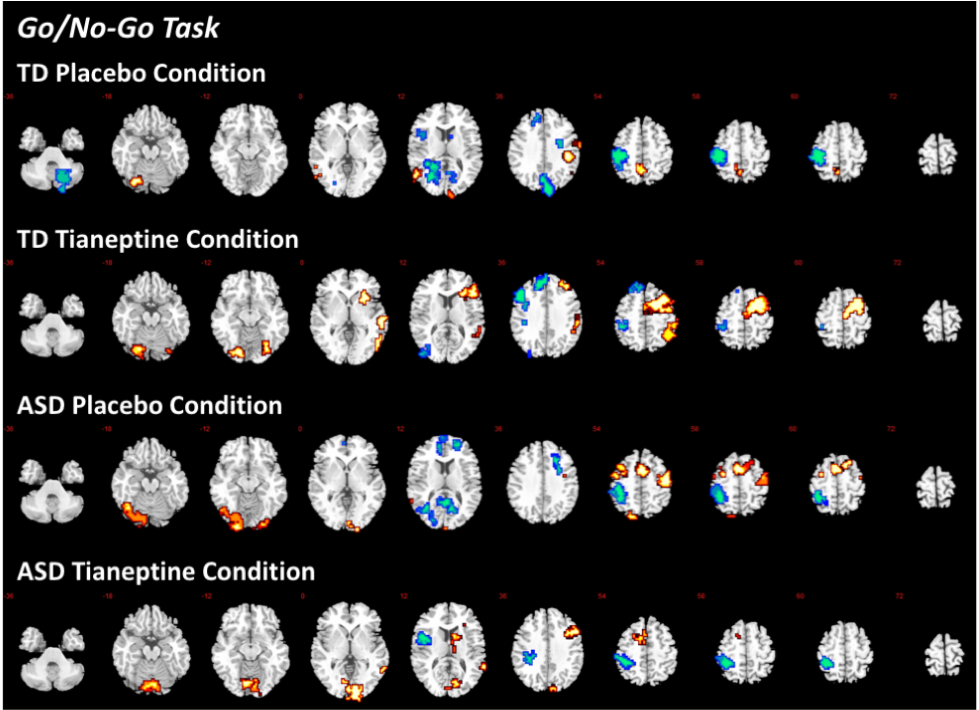


Locations of group-wise BOLD signals from No-Go versus Oddball. Red = No-Go > oddball. Blue = No-Go < oddball. Numeric label = z Talairach coordinate. Right hemisphere of brain is on the right side of the image. Abbreviations: BOLD, blood-oxygen-level dependent; TD, typically developed controls; ASD, individuals with autism spectrum disorder.

## Supplementary figure 2: Anatomical location and statistics for BOLD activation per group (ASD, TD) and drug (placebo, tianeptine) condition for the sustained attention task

*
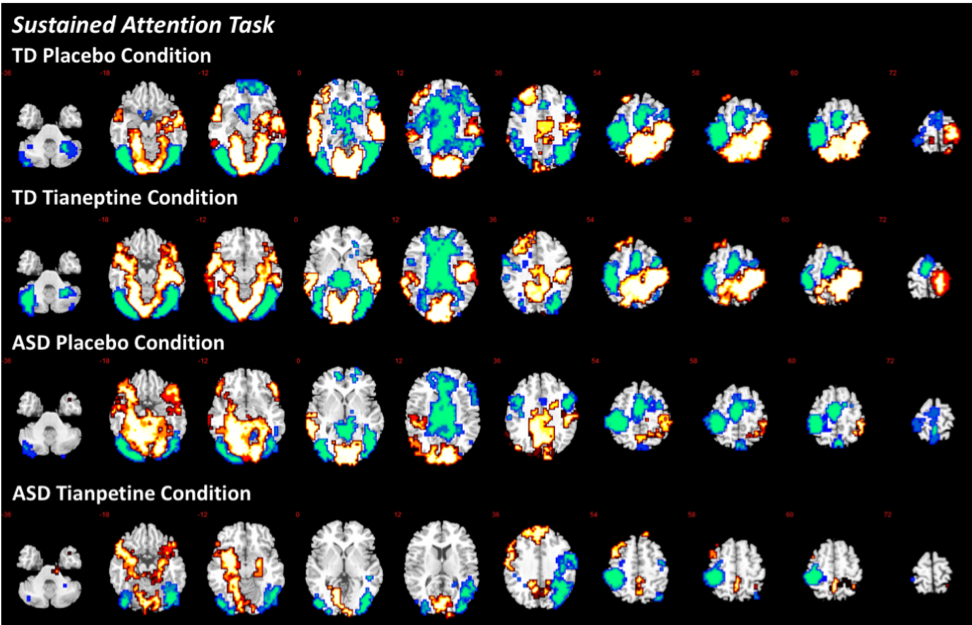
*

Locations of group-wise BOLD signals from 0.5s versus 8s contrasts. Red = 8s > 0.5s. Blue = 0.5s > 8s. Numeric label = z Talairach coordinate. Right hemisphere of brain is on the right side of the image. Abbreviations: BOLD, blood-oxygen-level dependent; TD, typically developed controls; ASD, individuals with autism spectrum disorder.

## References

1. Bullmore ET, Brammer MJ, Rabe-Hesketh S, Curtis VA, Morris RG, Williams SCR, et al. Methods for diagnosis and treatment of stimulus-correlated motion in generic brain activation studies using fMRI. Hum Brain Mapp. 1999;7(1):38-48.

2. Friman O, Borga M, Lundberg P, Knutsson H. Adaptive analysis of fMRI data. Neuroimage. 2003;19(3):837-45.

3. Bullmore E, Long C, Suckling J, Fadili J, Calvert G, Zelaya F, et al. Colored noise and computational inference in neurophysiological (fMRI) time series analysis: Resampling methods in time and wavelet domains. Hum Brain Mapp. 2001;12(2):61-78.

4. Bullmore ET, Suckling J, Overmeyer S, Rabe-Hesketh SA, Taylor EA, Brammer MJA. Global, voxel, and cluster tests, by theory and permutation, for a difference between two groups of structural MR images of the brain. Medical Imaging, IEEE Transactions on. 1999;18(1):32-42.

5. Talairach J, Tournoux P. Co-Planar Stereotaxic Atlas of the Human Brain: Three- Dimensional Proportional Systems. New York: Thieme Medical 1988.

6. Brammer MJ, Bullmore ET, Simmons A, Williams SCR, Grasby PM, Howard RJ, et al. Generic brain activation mapping in functional magnetic resonance imaging: A nonparametric approach. Magn Reson Imaging. 1997;15(7):763-70.
